# Lineage infidelity in FH-deficient RCC with secondary somatic alterations: a case report and implications for diagnosis and treatment

**DOI:** 10.1177/17588359261456676

**Published:** 2026-06-16

**Authors:** Jianping Zhao, Derek B. Allison, Giannicola Genovese, Stephanie Rock, Noah Spies, Michael Hensley, Gleb Khegai, Anastasiya Makeeva, Daria Melikhova, Lev Bedniagin, Patrick J. Hensley, Zin W. Myint, Pavlos Msaouel

**Affiliations:** Department of Anatomical Pathology, The University of Texas MD Anderson Cancer Center, Houston, TX, USA; Allison Department of Pathology and Laboratory Medicine, University of Kentucky College of Medicine, Lexington, KY 40536, USA; Department of Urology, University of Kentucky College of Medicine, Lexington, KY, USA Markey Cancer Center, Lexington, KY, USA; Department of Genitourinary Medical Oncology, The University of Texas MD Anderson Cancer Center, Houston, TX, USA; Department of Genomic Medicine, The University of Texas MD Anderson Cancer Center, Houston, TX, USA; David H. Koch Center for Applied Research of Genitourinary Cancers, The University of Texas, Anderson Cancer Center, Houston, MD, TX, USA; Caris Life Sciences, Phoenix, AZ, USA; Caris Life Sciences, Phoenix, AZ, USA; BostonGene Corporation, Waltham, MA, USA; BostonGene Corporation, Waltham, MA, USA; BostonGene Corporation, Waltham, MA, USA; BostonGene Corporation, Waltham, MA, USA; BostonGene Corporation, Waltham, MA, USA; Department of Pathology and Laboratory Medicine, University of Kentucky College of Medicine, Lexington, KY, USA; Department of Urology, University of Kentucky College of Medicine, Lexington, KY, USA; Markey Cancer Center, Lexington, KY, USA; Markey Cancer Center, Lexington, KY, USA; Division of Medical Oncology, Department of Internal Medicine, University of Kentucky, Lexington, KY, USA; Department of Genitourinary Medical Oncology, The University of Texas MD Anderson Cancer Center Unit 1374, 1155 Pressler St., Houston, TX 77030-3721, USA; David H. Koch Center for Applied Research of Genitourinary Cancers, The University of Texas Anderson Cancer Center, Houston, MD, USA; Department of Translational Molecular Pathology, The University of Texas MD Anderson Cancer Center, Houston, TX, USA; The University of Texas MD Anderson Cancer Center, UTHealth Houston Graduate School of Biomedical Sciences, Houston, TX, USA

**Keywords:** case report, fumarate hydratase-deficient renal cell carcinoma, hereditary leiomyomatosis, immunoradiotherapy, lineage infidelity, tumor evolution

## Abstract

Fumarate hydratase (FH)-deficient renal cell carcinoma (RCC) can exhibit striking histologic and immunophenotypic heterogeneity. A 36-year-old woman with uterine leiomyomas presented with a 5.1-cm left renal sinus mass and retroperitoneal adenopathy. Biopsy showed juxtaposed lower-grade oncocytic (PAX8 strong, GATA3 weak) and higher-grade pleomorphic (PAX8 negative, GATA3 and CK7 strong) components. DNA sequencing revealed germline pathogenic *FH* p.S419P, clonal *TP53* p.H193D, and subclonal *NF2* and *KMT2A* mutations, establishing FH-deficient RCC associated with hereditary leiomyomatosis and renal cell carcinoma syndrome. Nivolumab + cabozantinib reduced the lesion before nephrectomy, which showed predominantly pleomorphic tumor cells that were PAX8 negative and GATA3/p63 positive, mimicking upper tract urothelial carcinoma. Transcriptome sequencing mapped the tumor midway between renal and bladder cancer. Postoperatively, nivolumab + ipilimumab with radiotherapy achieved disease control. In this hypothesis-generating case report, *TP53*, *NF2*, and *KMT2A* secondary alterations on an *FH*-null background were associated with lineage infidelity, which can create diagnostic challenges that underscores the role for integrated pre‑ and post‑treatment multi‑omics in diagnostically ambiguous renal tumors.

## Plain language summary

When a rare inherited kidney cancer looks like bladder cancer: how integrated pathology and genetic testing helped guide diagnosis and treatment in one patient

Some rare kidney cancers can be very difficult to identify correctly. In this report, we describe a 36-year-old woman with an inherited form of kidney cancer linked to changes in the FH gene. This condition is associated with hereditary leiomyomatosis and renal cell carcinoma syndrome, which can also cause skin and uterine smooth muscle tumors. In her case, the kidney tumor did not look typical. Parts of it had features expected for kidney cancer, but other parts looked more like cancer of the urinary tract, also called urothelial or bladder-type cancer. This created a major diagnostic challenge. To clarify the diagnosis, the care team combined standard pathology with detailed DNA and RNA testing of tumor samples taken before and after treatment. These tests confirmed that the tumor was an FH-deficient renal cell carcinoma, even though the later surgical specimen had changed and closely resembled urothelial cancer. The molecular findings were especially helpful because treatment effects and inflammation made the post-treatment tissue harder to interpret. This distinction mattered clinically. Treatments commonly used for urothelial cancer were considered less appropriate after the molecular results showed that the tumor fit better with FH-deficient kidney cancer. Based on the full picture, the patient was treated with immunotherapy, targeted therapy against kidney cancer, surgery, and radiation. Her disease was controlled, and she remained clinically well at the latest follow-up described in the manuscript. This is a report of a single patient, so it cannot prove how often this happens or whether the additional mutations found in the tumor caused these unusual features. Even so, this case highlights an important practical lesson: when a kidney tumor has mixed or confusing features, especially in a younger patient, integrated pathology and molecular testing can be critical for making the correct diagnosis and choosing treatment.

## Introduction

Fumarate hydratase (FH)-deficient renal cell carcinoma (RCC) is a rare and aggressive subtype of RCC defined by pathogenic alterations of the *FH* gene, encoding FH, a key component of the tricarboxylic acid cycle.^1–3^ This entity can arise sporadically but is commonly associated with hereditary leiomyomatosis and renal cell carcinoma (HLRCC) syndrome, an autosomal dominant condition caused by germline *FH* mutations. Morphologically, FH-deficient RCCs often exhibit mixed features, including papillary, tubulocystic, and solid architectural patterns, and eosinophilic macronucleoli; however, some cases may have low-grade oncocytic morphology.^4–7^ These tumors often present at an advanced stage with early metastasis and poor prognosis. Diagnosis of this tumor relies on histopathologic examination and demonstration of germline/somatic mutation of *FH* gene by molecular testing or immunohistochemical evidence of FH loss and/or expression of S-(2-succino) cysteine (2SC), a sensitive marker in detecting defective FH enzyme function.^8,9^

Tumor lineage infidelity refers to the aberrant expression of cellular markers or differentiation features from lineages unrelated to a tumor’s tissue of origin.^10–13^ This phenomenon has been recognized in hematologic malignancies as well as a broad spectrum of solid tumors.^14–19^ From a diagnostic perspective, lineage infidelity poses a significant challenge for pathologists. Aberrant marker expression and divergent morphological features may lead to misclassification of tumor origin, particularly when limited biopsy samples are evaluated. Careful integration of histopathological, immunological, and molecular findings is often required to reach a correct diagnosis for tumors with lineage infidelity.^20,21^ Furthermore, recognition of lineage infidelity has emerging clinical relevance, as it may inform prognosis and influence therapeutic strategies, including the selection of lineage-specific targeted therapies or immunotherapies.^22^ Despite striking morphologic heterogeneity and prominent epithelial-to-mesenchymal transition consistent with lineage infidelity in FH-deficient RCC,^23–26^ published genomic, epigenomic, and transcriptomic studies to date have not shown lineage infidelity with consistent transcriptional rewiring toward extrarenal cell identities. Here, we presented an unusual case of FH-deficient RCC with lineage infidelity away from its original renal epithelial program.

## Case presentation

**Figure 1.**
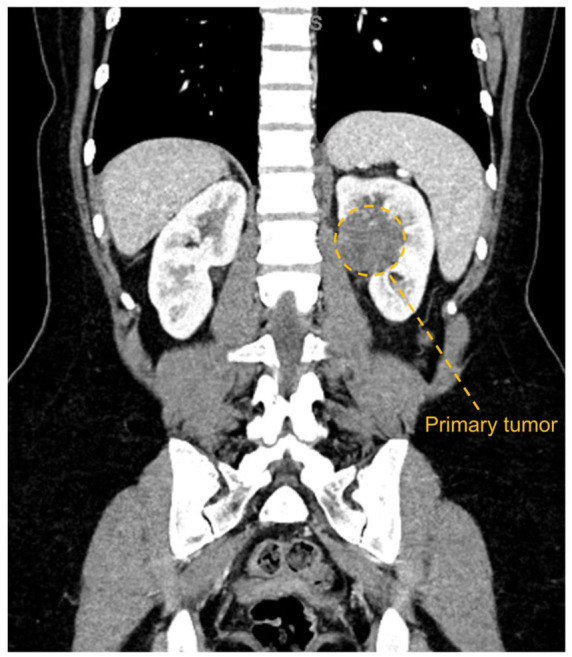
Coronal CT view of the primary left kidney tumor mass prior to nephrectomy.

**Figure 2.**
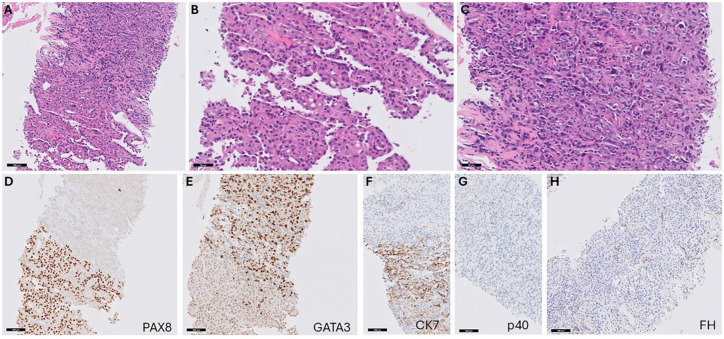
Histopathological evaluation of the renal mass biopsy specimen with immunohistochemical staining. (a) In this core, the lower portion contains an oncocytic component with variable architecture, while the upper portion shows a transition to a higher grade, pleomorphic component (H&E stain, 200× magnification, scale bar = 100 microns). (b) The oncocytic component at higher magnification shows abundant eosinophilic cytoplasm with variable vacuoles, as well as relatively monotonous nuclei with prominent nucleoli (H&E stain, 400× magnification, scale bar = 50 microns). (c) The pleomorphic component at higher magnification shows less eosinophilic features and significant nuclear pleomorphism with multinucleated tumor cells and admixed inflammatory cells (H&E stain, 400× magnification, scale bar = 50 microns). (d) PAX8 immunostain showing strong positivity in the oncocytic component while loss of staining is observed in the pleomorphic component (PAX8 stain, 200× magnification, scale bar = 100 microns). (e) GATA3 immunostain showing variable weak staining in the oncocytic component while strong staining is observed in the pleomorphic component (GATA3 stain, 200× magnification, scale bar = 100 microns). (f) CK7 immunostain showing positivity in the pleomorphic component (bottom of image) and focal weak to absent staining in the onocytic component (top of image; CK7 stain, 200× magnification, scale bar = 100 microns). (g) P40 imunostain showing a lack of staining the both the oncocytic and pleomorphic components (p40 stain, 200× magnification, scale bar = 100 microns). (h) FH immunostain showing a lack of staining in tumor cells with retained staining in vessels and inflammatory cells (FH stain, 200× magnification, scale bar = 100 microns). FH, fumarate hydratase.

The reporting of this study conforms to the CARE (CAse REport) Guidelines (see Supplemental File 1: CARE Checklist).^27^ A 36-year-old female patient had a history of uterine fibroids status post-uterine myomectomy at an outside hospital. She presented to the clinic for left flank pain. A 5.1-cm hypoenhancing left renal mass extending from the renal sinus was found on a CT scan, along with retroperitoneal lymphadenopathy (Figure 1). Biopsy of the renal mass revealed a solid tumor with two histological components, including a cribriforming and solid component with voluminous oncocytic cytoplasm and round nuclei with prominent nucleoli at 100× magnification (Figure 2). In these cores, however, there were multifocal adjacent areas displaying large, pleomorphic, hyperchromatic nuclei, including multinucleated forms with bizarre atypia and an infiltrative growth pattern with associated desmoplasia (Figure 2). Both components were intermixed and contained tumor-infiltrating lymphocytes with noticeably more inflammatory infiltration in the higher-grade pleomorphic component. These findings supported a singular clonal process, and tumor necrosis was identified. Immunohistochemical studies (Figure 2) showed that the complex oncocytic component was strongly positive for PAX8, weakly positive for GATA3, and negative for CK7. By contrast, the higher-grade, pleomorphic component was negative for PAX8, while strongly positive for GATA3 and CK7. Both components were negative for FH (FH deficient) and p40; SDHB was retained and TFE3 FISH was negative for *TFE3* gene rearrangement. To confirm the diagnosis, molecular testing (next-generation sequencing (NGS); whole exome and whole transcriptome sequencing, Caris Life Sciences (Irving, Texas, United States)) revealed an S419P mutation in the *FH* gene, along with pathogenic mutations in *KMT2A*, *NF2*, and *TP53*, discussed in greater detail below. Germline testing (Ambry Genetics CancerNext-Expanded^®^ + RNAinsight^®^ 71 gene panel) confirmed the S419P mutation to be in the germline. Subsequent dermatology evaluation confirmed the presence of skin leiomyomas with biopsies of the left proximal forearm, left mid-thigh, and left proximal lower leg, all consistent with leiomyoma cutis. Overall, the clinical and histological features of this tumor as well as ancillary studies supported the diagnosis of FH-deficient RCC associated with HLRCC syndrome.

**Figure 3.**
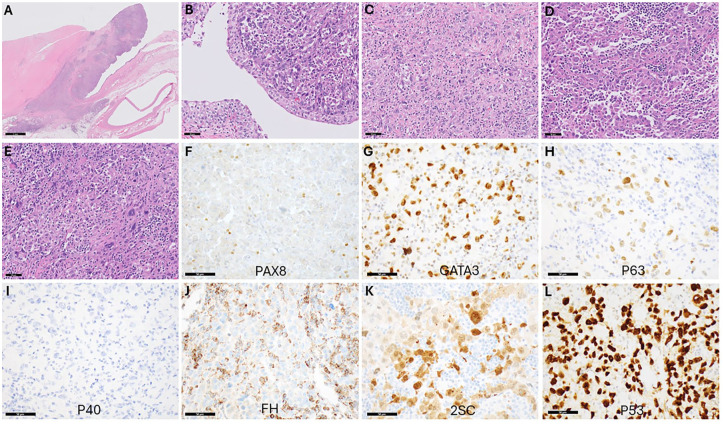
Histopathological evaluation of the nephrectomy sample with immunohistochemical staining. (a) Low power view of a large necrotic component of the tumor (left) immediately adjacent to a viable tumor that has protruded into the renal pelvis and is growing into the collecting system. This finding can mimic a renal pelvis urothelial carcinoma primary (H&E stain, 0.5× magnification, scale bar = 2 mm). (b) High power view showing an intact surface urothelium in the renal pelvis with pleomorphic carcinoma expanding the lamina propria. Note the lack of surface dysplasia (H&E stain, 200× magnification, scale bar = 50 microns). (c) Much of the tumor showed a relatively non-specific morphology with sheets and infiltrative tumor cells with a lack of oncocytic features and abundant mitotic activity. Much of the tumor showed a significant degree of infiltrative immune cells, which were predominantly lymphocytes (H&E stain, 200× magnification, scale bar = 50 microns). (d) Focal areas contained more oncocytic features, reminiscent of what was observed in parts of the biopsy (H&E stain, 200× magnification, scale bar = 50 microns). (e) Other areas displayed the pleomorphic features also seen in the biopsy (H&E stain, 200× magnification, scale bar = 50 microns). (f) PAX8 immunostain shows a lack of staining in tumor cells with weak staining in subsets of lymphocytes (PAX8 stain, 400× magnification, scale bar = 50 microns). (g) GATA3 immunostaining shows variably positive tumor cells, ranging from weak to strong (GATA3 stain, 400× magnification, scale bar = 50 microns). (h) A p63 immunostain shows weak and moderate staining in pleomorphic cells (p63 stain, 400× magnification, scale bar = 50 microns). (i) A p40 stain; however, shows a lack of staining (p40 stain, 400× magnification, scale bar = 50 microns). (j) A FH stain shows loss of staining in tumor cells with retained staining in lymphocytes, which can make it difficult to interpret (FH stain, 400× magnification, scale bar = 50 microns). (k) A 2SC stain shows variably positive staining in tumor cells (2SC stain, 400× magnification, scale bar = 50 microns). (l) A p53 stain shows strong diffuse nuclear positivity in tumor cells, consistent with the TP53 mutation identified on sequencing (p53 stain, 400× magnification, scale bar = 50 microns). 2SC, (2-succino) cysteine; FH, fumarate hydratase.

The patient was started on upfront systemic therapy with nivolumab and cabozantinib. After two cycles of therapy, imaging study showed that tumor size decreased to 3.2 cm with stable lymphadenopathy. Thereafter, open radical nephrectomy with regional and retrocrural lymphadenectomy was performed. The resection specimen revealed that the actual size of the infiltrative mass was 8.3 cm, involving the renal parenchyma, renal sinus, pelvicalyceal system, perinephric soft tissue, adrenal gland, and periadrenal soft tissue. There was marked tumor necrosis (~30%) present, consistent with a combination of high-grade tumor biology and treatment effect, based on the geographic distribution. Ten of 21 retroperitoneal and one of one retrocrural lymph nodes were positive for metastatic carcinoma. The perinephric soft tissue margin was focally positive for tumor with final pathology staged as ypT4/N1. Histologically, the tumor predominantly showed that the higher-grade pleomorphic component contained an infiltrative solid growth pattern, and only focally showed the oncocytic component with cribriforming, solid, and papillary-like patterns (Figure 3). All tumor cells exhibited prominent nucleoli. A panel of immunohistochemical stains was performed (Figure 3). Interestingly, unlike the original renal mass biopsy, the entire tumor on resection was negative for PAX2 and PAX8. On the other hand, the tumor was moderately positive for GATA3, focally positive for p63, and weakly positive for CK903, raising the possibility of a urothelial primary, while negative for uroplakin 2. The tumor-infiltrating lymphocyte component somewhat obscured the interpretation of the FH stain. For clarification, a 2SC immunostain was performed, but it showed variably positive staining (Figure 3) rather than the strong diffuse positivity typically found in FH-deficient RCC. The overall immunoprofile of the resection tumor was not clearly prototypical for FH-deficient RCC and brought up the possibility of a urothelial carcinoma, especially given the focal point of the mass abutting the renal pelvis, representing a diagnostic pitfall. Notably, pathogenic germline *FH* mutations have been occasionally noted in patients with upper tract urothelial carcinoma (UTUC),^28^ and radiologically both UTUC and FH-deficient RCC often appear as an infiltrative mass rather than a well-circumscribed, exophytic lesion (Figure 1).^29,30^

To elucidate the genomic and transcriptional features of this diagnostically challenging tumor, we integrated DNA- and RNA-based assays performed on both the pretreatment core biopsy and the post-treatment nephrectomy specimen. Comprehensive hybrid-capture NGS (whole exome and whole transcriptome sequencing; Caris Life Sciences) of the original renal mass biopsy specimen identified the pathogenic missense variant in *FH* (c.1255T>C; p.S419P) at a variant allele fraction (VAF) of 74%. Because non-neoplastic inflammatory and stromal elements comprised ~60% of the sample, the high VAF strongly supported biallelic inactivation of *FH* through mutation plus loss-of-heterozygosity (LOH). Additional somatic alterations included *TP53* c.577C>G (p.H193D) at a VAF of 58%, as well as *NF2* c.600-1G>A (canonical splice-site mutation) and a truncating *KMT2A* c.4647G>A (p.W1549*) mutation both at lower VAFs (9% and 10%, respectively), indicating subclonal acquisition. No pathogenic variants were detected in *SDHB*, *SDHC*, *TFE3*, *TSC1/2*, *VHL*, or other RCC-defining genes. The tumor mutational burden (TMB) was 3 mut/Mb, and the microsatellite status was stable. LOH was noted in 15% of tested genomic segments.

**Figure 4.**
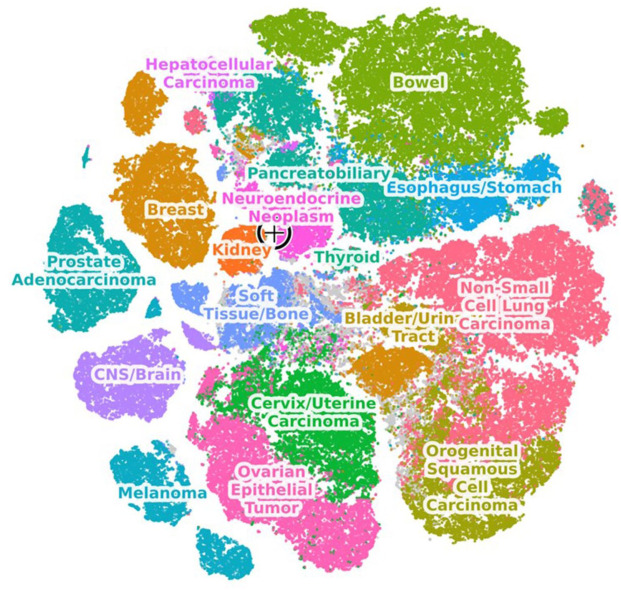
Transcriptomic profiling of the pretreatment biopsy sample. RNA-seq was used to obtain gene expression profiles of tumor samples. A global embedding of cancer types was calculated by transforming these gene expression profiles using PCA, followed by a t-SNE transformation to obtain a 2-dimensional approximate “map” of transcriptional similarity. The new patient sample is projected onto a pre-calculated PCA embedding space based on historical samples analyzed at Caris. Its proximity to other samples on the map reflects shared transcriptional programs with these samples. Only the immediate neighborhoods of samples should be considered for further interpretation since t-SNE embeddings do not preserve similarity correlations over long distances. Each point on the t-SNE map represents a different sample. The target symbol represents the present patient’s pretreatment biopsy sample. PCA, principal component analysis; t-SNE, t-stochastic neighbor embedding.

Projection of RNA-seq from the pretreatment core biopsy onto the Caris pan-cancer transcriptomic map revealed that the sample lay outside the compact cluster formed by canonical kidney tumors (Figure 4). Although the biopsy contained both the complex oncocytic component and the higher-grade pleomorphic component, their combined transcriptome plots occupied an “interstitial” space whose nearest neighbors were extrarenal cancers rather than other RCCs. Given that t-stochastic neighbor embedding (t-SNE) preserves only local relationships, global distances should not be over-interpreted; nonetheless, the conspicuous lack of adjacent RCC samples in the immediate neighborhood indicates that lineage infidelity was already apparent at diagnosis, preceding the more pronounced loss of kidney differentiation documented after systemic therapy.

**Figure 5.**
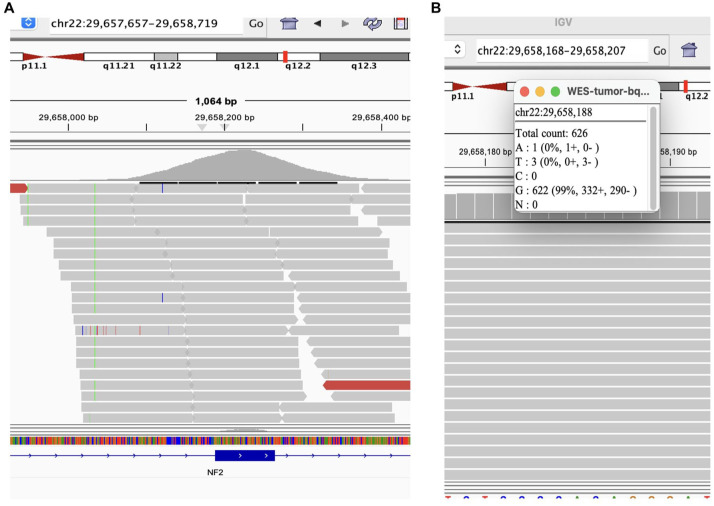
Sequencing coverage at the NF2 c.600-1G>A splice-site locus in the post-treatment nephrectomy specimen. (a) IGV screenshot of whole-exome sequencing reads at chr22:29,657,657–29,658,719, showing the NF2 gene (bottom track) and the canonical splice-site position (chr22:29,658,188). (b) Allele-level read counts at chr22:29,658,188: of 626 total reads, 622 (99%) support the reference allele (G) and only 1 read carries the variant allele (A), well below validated detection thresholds. The near-absence of variant-supporting reads at high coverage depth indicates that the NF2 c.600-1G>A subclone identified in the pretreatment biopsy (VAF 9%) was no longer detectable in the post-treatment specimen, consistent with clonal loss under treatment-related selective pressure. IGV, integrative genomics viewer; VAF, variant allele fraction.

Whole exome and transcriptome sequencing of the post-treatment nephrectomy specimen was performed by BostonGene Corporation as previously described.^18^ Histology-guided macro-dissection revealed a tumor fraction <20%, preventing high-confidence somatic calling; nevertheless, low-depth exome data corroborated the presence of the germline *FH* S419P mutation and confirmed retention of the core-biopsy *TP53* p.H193D mutation (VAF 27.5%) and of the likely subclonal truncating *KMT2A* p.W1549* mutation (VAF 6.7%). In contrast, the NF2 c.600-1G>A splice-site variant was not detected despite adequate coverage at the locus (626× depth), with only 1 of 626 reads carrying the variant allele (Figure 5). Had the subclone persisted at its pretreatment frequency (~9% VAF), approximately 56 variant-supporting reads would have been expected at this depth, indicating that the NF2-mutant subclone was lost or reduced below the limit of detection, likely through clonal evolution or treatment-related selection.

**Figure 6.**
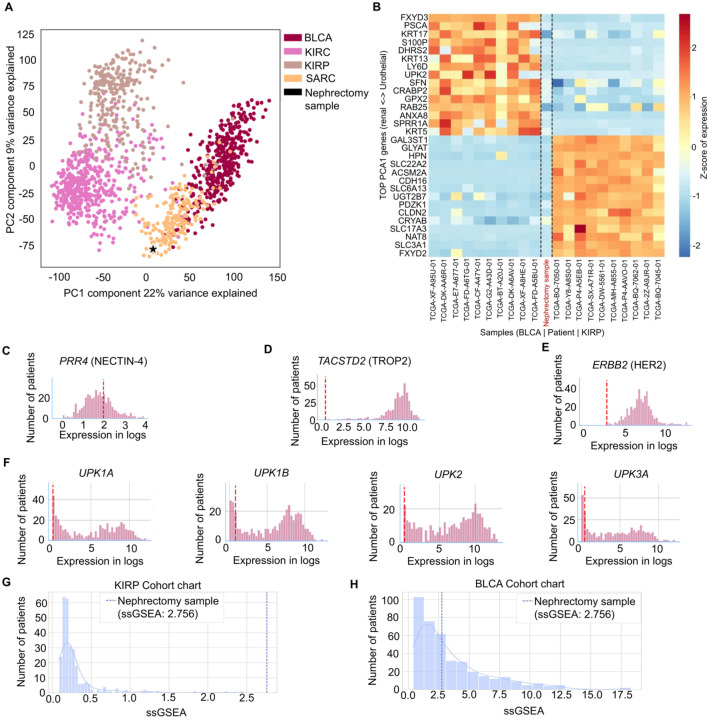
Transcriptomic profiling of the nephrectomy sample. (a) PCA of batch-corrected RNA-seq expression data (Procrustes model) for the nephrectomy sample (black star) projected alongside urothelial carcinoma of the bladder (BLCA), clear cell renal cell carcinoma (KIRC), papillary renal cell carcinoma (KIRP), and SARC from the Cancer Genome Atlas (TCGA). (b) Heatmap of the top genes contributing to PC1, displayed as *Z*-scores of expression for representative BLCA (left) and KIRP (right) TCGA samples with the nephrectomy sample (center, red label). Genes are ordered by PC1 loading, from urothelial identity markers (top) to renal tubular identity markers (bottom). (c–e) Distribution of *PRR4* (c), *TACSTD2* (d), and *ERBB2* (e) transcript expression in the TCGA BLCA cohort (purple bars) compared with the nephrectomy sample (red dashed line). (f) Expression of uroplakin genes (*UPK1A*, *UPK1B*, *UPK2*, *UPK3A*) in the TCGA BLCA cohort with the nephrectomy sample indicated as in (c–e), showing near-undetectable expression of downstream urothelial structural proteins. (g, h) ssGSEA enrichment scores for an EMT gene signature in the TCGA KIRP cohort (g) and TCGA BLCA cohort (h); the nephrectomy sample (dashed blue line, ssGSEA = 2.756) is a marked outlier relative to KIRP, consistent with EMT-driven dedifferentiation, while falling within the lower range of BLCA. EMT, epithelial–mesenchymal transition; PCA, principal component analysis; SARC, sarcoma; ssGSEA, single-sample gene set enrichment analysis.

RNA-seq data of the post-treatment nephrectomy specimen were batch-corrected using the Procrustes model, which transforms FFPE exome-capture RNA expression profiles into polyA-protocol expression space, enabling direct comparison with fresh-frozen TCGA cohorts.^31^ Dimensionality reduction with principal component analysis (PCA) was subsequently applied (Figure 6(a)) relative to clear cell RCC (KIRC), papillary RCC (KIRP), urothelial carcinoma of the bladder (BLCA), and sarcoma (SARC) from the Cancer Genome Atlas (TCGA).^31^ Since the nephrectomy tissue predominantly lacked overt FH-deficient RCC histology and was more homogeneous, we opted for a PCA instead of another t-SNE projection to quantify how far the resection specimen had moved away from bona fide renal-lineage expression space. Strikingly, the sample mapped equidistant between canonical bladder cancer and RCC clusters, rather than clustering with either cohort, underscoring a transcriptional “hybrid” identity consistent with immunophenotypic lineage infidelity. Analysis of the top genes contributing to PC1 (22% variance explained) confirmed that the separation is driven by lineage-specific epithelial differentiation markers—urothelial identity genes (e.g., *FXYD3*, *PSCA*, *KRT17*, *UPK2*) versus renal tubular identity genes (e.g., *CDH16*, *SLC3A1*, *FXYD2*)—rather than immune, stromal, or housekeeping transcripts (Figure 6(b)). The nephrectomy sample showed low expression of both urothelial and renal gene sets, consistent with erosion of both lineage programs. Despite its urothelial-like GATA3/p63 expression and moderate levels of *PRR4* (NECTIN-4) transcripts (Figure 6(c)), the tumor showed near-undetectable transcript levels of other urothelial therapeutic targets such as *TACSTD2* (TROP2; Figure 6(d)), and *ERBB2* (HER2; Figure 6(e)). IHC for HER2 confirmed lack of expression (HER2 score = 0).

To assess whether the tumor had executed a full urothelial differentiation program beyond upstream transcription factor expression, we interrogated RNA-seq data for downstream urothelial-specific structural proteins. Expression of *UPK1A*, *UPK1B*, *UPK2*, and *UPK3A* was near-undetectable in the nephrectomy sample, well below the TCGA BLCA distribution for each gene (Figure 6(f)). Single-sample gene set enrichment analysis (ssGSEA) using an EMT gene signature yielded an enrichment score of 2.756, placing the sample as a marked outlier relative to the TCGA KIRP cohort (Figure 6(g)) but within the lower range of the TCGA BLCA distribution (Figure 6(h)). The absence of downstream uroplakin expression combined with elevated EMT enrichment indicated that the tumor’s departure from renal identity reflected EMT-associated dedifferentiation with partial adoption of urothelial transcription factors rather than complete urothelial lineage reprogramming.

## Outcome and follow-up

Within 4 months of surgery, the patient developed metabolically active nodal metastases in the left supraclavicular fossa, right pulmonary hilum, and retrocrural space that progressed despite resumption of cabozantinib in combination with nivolumab. Because the tumor showed unequivocal biallelic FH loss together with a low TMB, unlike the high mutational burden that usually characterizes UTUC (median TMB of 13.2 mut/Mb with interquartile range of 7.4–19.1 mut/Mb),^32^ these findings confirmed the recurrence as FH-deficient RCC rather than urothelial carcinoma. Crucially, while moderate *PRR4* (NECTIN-4) levels did not rule out the potential use of enfortumab vedotin, RNA-seq showed minimal transcript levels of *TACSTD2* (TROP2), and *ERBB2* (HER2), eliminating the therapeutic rationale for sacituzumab govitecan or HER2-directed regimens used in metastatic urothelial cancer. In addition, cabozantinib resistance made continued VEGFR/MET inhibition unattractive, whereas the durable responses to dual checkpoint blockade reported in papillary-like RCC with nivolumab + ipilimumab plus radiotherapy offered a biologically consonant alternative.^33^

**Figure 7.**
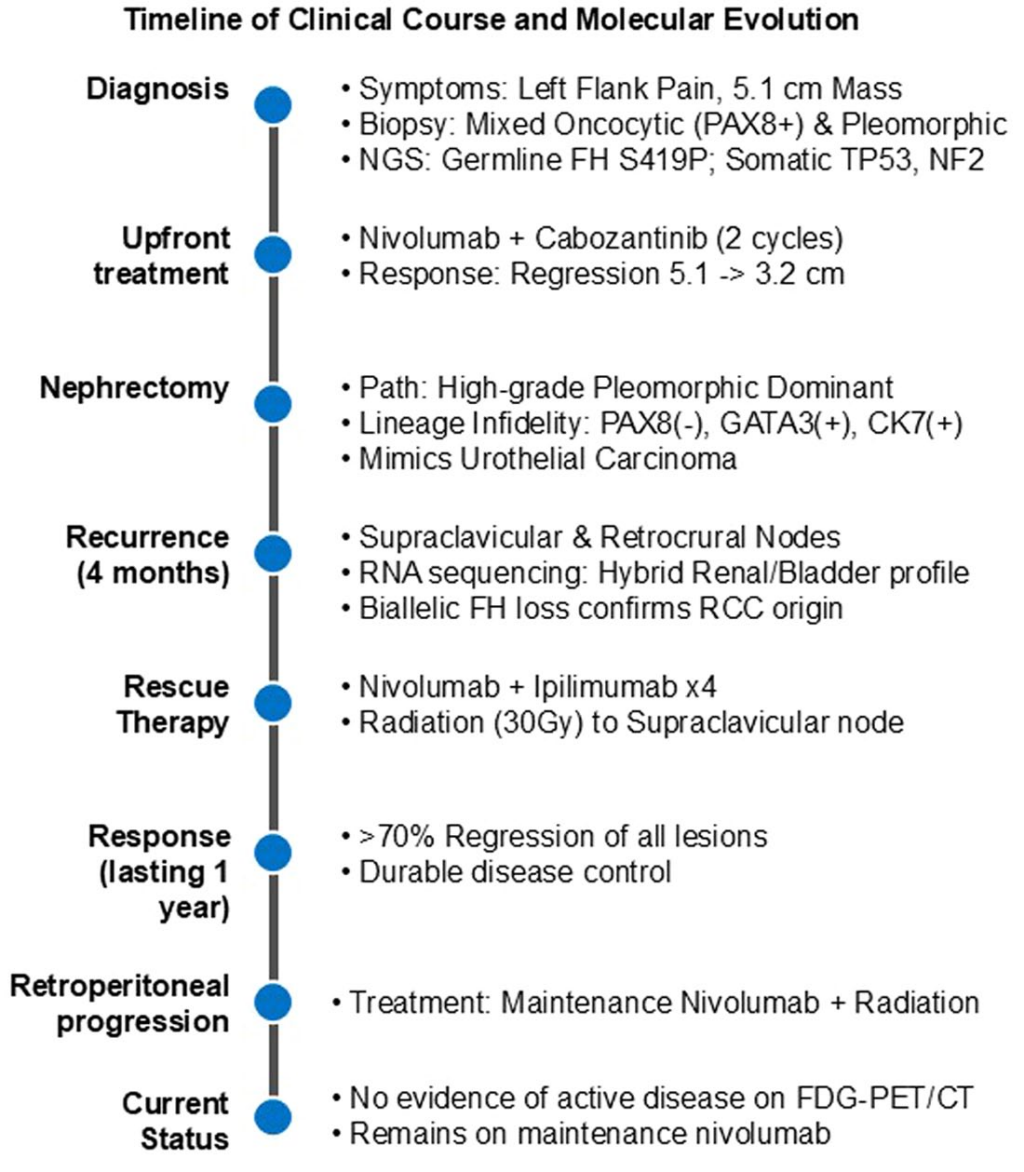
Timeline of the patient’s clinical course.

Accordingly, we initiated nivolumab 3 mg/kg and ipilimumab 1 mg/kg every 3 weeks for four doses, followed by maintenance nivolumab, integrated with radiation therapy to the painful left supraclavicular lymphadenopathy (30 Gy in five fractions). This treatment strategy resulted in marked regression (>70% diameter reduction) of all treated and untreated lesions with no new sites of disease, and the patient remained radiographically and clinically well for 1 year after nephrectomy. She subsequently developed progressive retroperitoneal adenopathy treated with further radiation therapy while continuing maintenance nivolumab with excellent tolerance (Figure 7). The patient remains on maintenance nivolumab with no evidence of disease activity on fluorodeoxyglucose positron emission tomography/CT imaging at the last follow-up 16 months after nephrectomy.

## Discussion

The germline *FH* p.S419P variant with copy-neutral LOH represents the obligate initiating lesion in this carcinoma. Complete loss of FH enzymatic activity leads to intra-mitochondrial accumulation of fumarate, which subsequently diffuses to the cytosol, where it modifies cysteine residues through succination (2SC) and competitively inhibits α-ketoglutarate-dependent dioxygenases, such as prolyl-hydroxylases and TET/JKDM family chromatin regulators.^8,34^ These alterations stabilize HIF1α, enforce the NRF2 oxidative-stress program, and create a hyper-succinated epigenetic landscape that primes cells for mesenchymal transition and early metastatic proclivity.^8,35,36^ In our patient, clonal *TP53* p.H193D loss coincided with elevated LOH, supporting the paradigm that defective p53 surveillance accelerates chromosomal instability in FH-deficient RCC. Subclonal *NF2* splice-site (c.600-1G>A) and truncating *KMT2A* p.W1549* mutations likely emerged later. Functional studies in murine models demonstrate that merlin (NF2) loss cooperates with fumarate accumulation to activate YAP/TAZ-mediated EMT,^37,38^ whereas KMT2A inactivation suppresses H3K4 trimethylation and disrupts HOX-controlled differentiation,^39^ together fostering the pleomorphic sarcomatoid phenotype frequently observed in advanced FH-deficient RCC.^40^ The combined genomic hits may have translated into a transcriptional identity crisis. RNA-seq of the nephrectomy mapped midway between TCGA RCC and urothelial carcinoma clusters, and immunohistochemistry showed gain of GATA3/p63 with loss of PAX8. Importantly, however, this lineage infidelity was partial rather than complete: downstream urothelial structural effectors (uroplakins) were near-undetectable, indicating that the tumor acquired upstream urothelial transcription factor expression without engaging the terminal urothelial differentiation program. ssGSEA using an EMT gene signature revealed that the nephrectomy sample’s EMT enrichment score dramatically exceeded that of any KIRP sample while falling within the lower range of BLCA, consistent with EMT-driven dedifferentiation as a mechanism for lineage erosion. This is concordant with the subclonal *NF2* mutation, whose protein product merlin normally suppresses YAP/TAZ-mediated EMT,^37,38^ and with the pleomorphic sarcomatoid morphology that dominated the resection specimen. Together, these findings suggest that the tumor eroded its renal epithelial identity through EMT-associated dedifferentiation and partially co-opted upstream elements of the urothelial transcriptional program, creating a lineage-ambiguous state sufficient to mimic urothelial carcinoma diagnostically without completing a full lineage switch. Such partial lineage infidelity echoes broader observations that sustained oncogenic stress can erode lineage-restrictive chromatin barriers, allowing tumors to adopt marker repertoires of unrelated epithelia without necessarily completing the full differentiation cascade.^10–13^ While aberrant cytokeratin or hormone-receptor expression is well documented in other solid tumors,^14–17^ to our knowledge, lineage infidelity toward a urothelial program has not been reported at the molecular level in FH-deficient RCC.

In a large series of FH-deficient RCCs, focal p63 expression was identified in 1 of 29 cases (3.4%), while strong p63 staining was also observed in only 1 of 29 cases (3.4%).^41^ Notably, the case with focal p63 expression also demonstrated weak GATA3 positivity. Within that cohort, weak to focal GATA3 staining was present in 11 of 29 cases (37.9%), with an additional case showing weak staining (3.4%) and 2 cases (6.9%) demonstrating strong GATA3 expression. In contrast, PAX8 immunohistochemistry, when performed, was strongly positive in the majority of cases (24/26; 92.3%), while the remaining two cases (7.7%) exhibited only weak staining. Furthermore, CK7 expression was exceedingly rare, with only 1 of 33 tumors (3.0%) demonstrating positivity, mirroring the immunophenotype of the current case.

While this study suggests that a subset of FH-deficient RCCs may exhibit partial immunophenotypic deviation from canonical renal epithelial lineage markers, sequencing was limited to the *FH* gene and did not investigate the potential contribution of secondary genomic alterations or transcriptional profiles. Follow-up studies have corroborated these findings; one series reported focal weak-to-strong GATA3 expression in 7 of 11 cases (63.6%), though all remained CK7-negative.^42^ Among them, one case harboring secondary mutations in NF2, LZTR1, B2M, MAP3K4, and FRS1 displayed diffuse GATA3 staining, although morphologic details were not provided.

In this hypothesis-generating single-patient case, secondary somatic alterations in TP53, NF2, and KMT2A may have co-occurred with clonal evolution toward a high-grade component with immunophenotypic and transcriptomic evidence of lineage infidelity. However, this observation is inferential and does not establish whether these alterations causally mediated transdifferentiation, clonal selection, therapy-associated phenotypic drift, or some combination thereof. While morphologic divergence from expected patterns has been described in other hereditary RCC syndromes, most notably SDH-deficient RCC,^43,44^ this present case is, to our knowledge, the first reported instance of FH-deficient RCC exhibiting a combined loss of the renal lineage factor PAX8 with acquired expression of CK7, GATA3, and p63, strongly mimicking urothelial carcinoma.

Systemic treatment with nivolumab plus cabozantinib appears to have debulked the lower-grade oncocytic, PAX8-positive component but spared a PAX8-negative *TP53*/*KMT2A*-mutant clone that ultimately dominated the post-treatment specimen and all subsequent metastases. These findings are compatible with treatment-associated clonal selection, although sampling differences and therapy-related phenotypic drift cannot be excluded. Systemic therapies can sculpt clonal architecture and unintentionally enrich adaptive or lineage-ambiguous populations.^45^ A practical clinical lesson from this case is that post-treatment tissue can become diagnostically misleading, particularly in heavily treated tumors with prominent inflammation, fibrosis, necrosis, and therapy effect; in such settings, pretreatment biopsy material integrated with molecular findings may provide the most reliable basis for classification.

Radiologically, the sinus-centered mass and pelvicalyceal involvement, coupled with diffuse GATA3/CK7 expression and equivocal FH/2SC staining after therapy, closely mimicked UTUC. Isolated reports of germline *FH* mutations in UTUC further complicated the picture; however, biallelic inactivation, as seen in this present case, have not been reported in urothelial carciomas.^28^ Our experience highlights the perils of relying on a single time-point biopsy or post-treatment specimen for lineage assignment in tumors with high plasticity. Comprehensive molecular profiling of pretreatment tissue proved decisive, anchoring the diagnosis to FH-deficient RCC and averting potentially ineffective urothelial-directed regimens such as sacituzumab govitecan, for which the tumor lacked TROP2 transcript expression.

Treatment selection for metastatic FH-deficient RCC remains empirical.^22,46^ Vascular-directed TKIs yield responses in a subset, but resistance is common. Dual checkpoint blockade with nivolumab plus ipilimumab has achieved durable regressions in non-clear-cell and papillary-like RCC, including a seminal case that achieved a complete metabolic response when combined with stereotactic radiotherapy,^33^ as well as encouraging activity in the prospective CheckMate-920 non-clear cell cohort.^47^ In phase II testing, nivolumab plus cabozantinib produced objective responses in 15 out of 32 (47%) cases of papillary RCC,^48^ supporting the frontline use of this combination when rapid cytoreduction is required.

As a single-patient case report, this study is inherently hypothesis-generating, limits generalizability, and does not permit statistical testing, cohort-level validation, or causal inference regarding the relationship between secondary alterations and lineage-infidelity features in FH-deficient RCC. We also acknowledge the potential for publication bias, as unusual and diagnostically complex cases such as this are more likely to come to publication and therefore should not be interpreted as evidence of frequency. The prevalence of confirmed lineage infidelity in FH-deficient RCC remains unknown.

Because our transcriptomic analysis was based on bulk RNA-seq, we cannot determine whether the observed findings reflect true transdifferentiation versus clonal replacement/selection. Furthermore, fumarate flux or post-treatment succination burden were not quantified and the interpretation of metabolic persistence after therapy was therefore indirect and based on pathologic and genomic context rather than direct metabolomic measurement. Functional validation of TP53/NF2/KMT2A cooperation in the FH-deficient context is still lacking, as are metabolomic data to quantify oncometabolite flux after systemic therapy. Future investigations should couple single-cell multi-omics with spatial transcriptomics in larger FH-deficient RCC cohorts to decipher the epigenetic circuits that permit extrarenal lineage infidelity and to identify vulnerabilities that could be therapeutically exploited. Review of the raw sequencing data revealed that the NF2 splice-site locus had adequate coverage (626×) in the nephrectomy specimen, yet the variant was supported by only 1 of 626 reads, indicating probable loss of the NF2-mutant subclone under treatment-related selective pressure rather than a technical detection failure. Lack of serial circulating-tumor DNA prevented temporal mapping of the clonal dynamics underlying this differential subclonal fate. The diagnostic resolution achieved here relied on specialized integrated pathology and molecular profiling that may not be universally available. However, the present case remains clinically useful because it highlights a practical diagnostic principle: when renal and urothelial features are discordant, especially in a younger patient or in a tumor with unusual morphology, FH-deficient RCC should remain in the differential and may warrant referral for expert pathology review and molecular testing. Notably, the observed clinical benefit cannot be attributed to any single treatment modality. Follow-up remains limited, and the long-term durability of the ongoing response is unknown. The intent of the present report is not to assign efficacy to one intervention, but rather to describe how integrated diagnosis-informed management operates in a therapeutically complex setting. In renal tumors with discordant renal and urothelial features, particularly when the morphology is unusual, or the patient context raises suspicion for hereditary disease, integrated pretreatment histology, molecular profiling, and germline evaluation should be considered before anchoring on urothelial carcinoma-directed treatment paradigms.

## Conclusion

In conclusion, this single-patient case illustrates how sequential genomic hits on an FH-deficient background may contribute to lineage infidelity, confound routine diagnostics, and modulate therapeutic vulnerabilities. Integrating histology with pre- and post-treatment multi-omics not only resolved the diagnostic dilemma but also guided a therapeutic strategy that achieved disease control. As comprehensive profiling becomes routine, deciphering the molecular logic of lineage infidelity promises to refine the management of rare, metabolically driven renal tumors.

## Supplemental Material

sj-docx-1-tam-10.1177_17588359261456676 – Supplemental material for Lineage infidelity in FH-deficient RCC with secondary somatic alterations: a case report and implications for diagnosis and treatment Supplemental material, sj-docx-1-tam-10.1177_17588359261456676 for Lineage infidelity in FH-deficient RCC with secondary somatic alterations: a case report and implications for diagnosis and treatment by Jianping Zhao, Derek B. Allison, Giannicola Genovese, Stephanie Rock, Noah Spies, Michael Hensley, Gleb Khegai, Anastasiya Makeeva, Daria Melikhova, Lev Bedniagin, Patrick J. Hensley, Zin W. Myint and Pavlos Msaouel in Therapeutic Advances in Medical Oncology

## Declarations

**Ethics approval and consent to participate:** The study was conducted in accordance with the Declaration of Helsinki and was approved by the Institutional Review Board of The University of Texas MD Anderson Cancer Center (protocol #2025-0949) and the University of Kentucky College of Medicine (protocol #79922). This project has received a waiver of informed consent and authorization from the University of Texas MD Anderson Cancer Center Institutional Review Board (protocol #2025-0949).

**Consent for publication:** The patient was informed and provided explicit consent for publication of the clinical data reported in this case report. All data have been sufficiently anonymized to protect patient privacy.

**Competing interests:** P.M. has received honoraria for service on a Scientific Advisory Board for Mirati Therapeutics, Bristol Myers Squibb, and Exelixis; consulting for Axiom Healthcare Strategies; non-branded educational programs supported by DAVA Oncology, Exelixis, and Pfizer; and research funding for clinical trials from Regeneron Pharmaceuticals, Takeda, Bristol Myers Squibb, Summit Therapeutics, Merck, Mirati Therapeutics, Gateway for Cancer Research, and the University of Texas MD Anderson Cancer Center. M.H., G.K., A.M., D.M., and L.B. are employees of BostonGene, Corporation. S.R. and N.S. are employees of Caris Life Sciences. The remaining authors declare no competing interests.

**Availability of data and materials:** All results generated and analyzed during the study are included in this published article. Raw data are not provided due to patient privacy concerns. Requests to access raw data should be forwarded to the corresponding author. This paper does not report original code. Any additional information required to reanalyze the data reported in this paper is available from the corresponding authors* upon request.
