# Supplementary material for: Lineage infidelity in FH-deficient RCC with secondary somatic alterations: a case report and implications for diagnosis and treatment
Source: Ther Adv Med Oncol. 2026 Jun 16;18:17588359261456676. doi: 10.1177/17588359261456676 (PMC13272986; doi:10.1177/17588359261456676)
Supplement: sj-docx-1-tam-10.1177_17588359261456676 – Supplemental material for Lineage infidelity in FH-deficient RCC with secondary somatic alterations: a case report and implications for diagnosis and treatment [file sj-docx-1-tam-10.1177_17588359261456676.docx]

**Supplementary File 1**

**CARE Checklist**

CARE Checklist for: *Lineage Infidelity in FH-Deficient RCC with Secondary Somatic Alterations: A Case Report and Implications for Diagnosis and Treatment*

| **Topic** | **CARE Checklist Item** | **Location** | **Reported Information** |
| --- | --- | --- | --- |
| **Title** | The diagnosis or intervention of primary focus followed by the words “case report” | Title page | Title includes “Case Report” and primary diagnosis (FH-Deficient RCC with Lineage Infidelity) |
| **Keywords** | 2–5 key words identifying diagnoses or interventions | Title page | Case report; Fumarate hydratase-deficient renal cell carcinoma; Lineage infidelity; Hereditary leiomyomatosis; Immunoradiotherapy; Tumor evolution |
| **Abstract** | Introduction, case presentation summary, and conclusion in structured or unstructured format | Abstract | Structured abstract with background (FH-deficient RCC), case summary (36-year-old woman, molecular findings, treatment course), and conclusion (multi-omics resolved diagnosis) |
| **Introduction** | Brief background summary and case presentation rationale | Introduction | Background on FH-deficient RCC, HLRCC syndrome, and lineage infidelity; rationale for reporting this novel phenotype |
| **Patient Information** | Demographics, main symptoms, medical/family/psychosocial history, relevant comorbidities | Case presentation | 36-year-old woman; left flank pain; history of uterine fibroids s/p myomectomy; germline FH mutation (HLRCC syndrome); cutaneous leiomyomas confirmed on dermatologic evaluation |
| **Clinical Findings** | Key findings from physical examination | Case presentation | Left flank pain; 5.1-cm renal sinus mass on CT; retroperitoneal lymphadenopathy; skin leiomyomas on dermatologic exam |
| **Timeline** | Historical and current information organized as a timeline | Case presentation; Outcome; Figure 6 | Chronological narrative: presentation → biopsy → nivolumab/cabozantinib → nephrectomy → recurrence → nivolumab/ipilimumab + RT → 1-year disease control → subsequent progression managed with RT. Figure 6 provides clinical timeline |
| **Diagnostic Assessment** | Diagnostic methods, diagnostic challenges, diagnostic reasoning, prognostic characteristics | Case presentation; Discussion | IHC panel (PAX8, GATA3, CK7, FH, 2SC, p63, p40, SDHB, TFE3); WES and WTS (Caris Life Sciences and BostonGene); germline testing (Ambry Genetics); diagnostic challenge of urothelial mimicry resolved by integrated multi-omics |
| **Therapeutic Intervention** | Types of intervention, administration, duration | Case presentation; Outcome | Nivolumab + cabozantinib (2 cycles, neoadjuvant); radical nephrectomy with lymphadenectomy; nivolumab 3 mg/kg + ipilimumab 1 mg/kg q3w × 4 then maintenance nivolumab; RT 30 Gy/5 fractions to supraclavicular nodes; subsequent RT to retroperitoneal adenopathy |
| **Follow-up and outcomes** | Clinician-assessed and patient-assessed outcomes, important follow-up test results, intervention adherence/tolerability, adverse events | Outcome | >70% diameter reduction of treated and untreated lesions; disease control at 1 year post-nephrectomy; excellent tolerance; subsequent retroperitoneal progression managed with further RT and maintenance nivolumab |
| **Discussion** | Strengths and limitations, relevant medical literature, rationale for conclusions, main takeaway lessons | Discussion | Strengths: integrated pre/post-treatment multi-omics, two independent sequencing platforms. Limitations: single case, bulk RNA-seq, low tumor purity post-treatment, no functional validation. Literature on FH-deficient RCC lineage markers contextualized. Lessons: diagnostic pitfall of urothelial mimicry; negative biomarker utility of transcriptomics |
| **Patient Perspective** | Patient sharing their perspective on treatments received | N/A | Not obtained (IRB waiver of informed consent) |
| **Informed Consent** | Patient informed consent documented | Declarations | Waiver of informed consent and authorization granted by MD Anderson Cancer Center IRB (protocol #2025-0949) |

Reference: Riley DS, Barber MS, Kienle GS, et al. CARE guidelines for case reports: explanation and elaboration document. *J Clin Epidemiol* 2017; 89: 218-235.
